# Analysis of World Championship Swimmers Using a Performance Progression Model

**DOI:** 10.3389/fpsyg.2019.03078

**Published:** 2020-01-22

**Authors:** Inmaculada Yustres, Jesús Santos del Cerro, Fernando González-Mohíno, Michael Peyrebrune, José María González-Ravé

**Affiliations:** ^1^Department of Physical Activity and Sport Sciences, University of Castilla-La Mancha, Toledo, Spain; ^2^Facultad de Lenguas y Educación, Universidad Nebrija, Madrid, Spain; ^3^Department of Statistics, University of Castilla-La Mancha, Toledo, Spain; ^4^School of Sport, Exercise and Health Sciences, Loughborough University, Loughborough, United Kingdom

**Keywords:** analysis, data, performance, talent, youth

## Abstract

**Purpose:**

The primary aim was to create a performance progression model of elite competitors in the World Swimming Championships from 2006 to 2017 for all strokes and distances. Secondly, to identify the influence of annual ratios of progression, ages of peak performance and junior status on success in senior competitions.

**Methods:**

Data regarding the participants of senior and junior World Championships (WCs) between 2006 and 2017 were obtained from FINA. The final filtered database, after removing those swimmers who just participated in junior WCs, included 4076. Statistical models were used to examine differences between the top senior swimmers (the top 30% best performances; T30) and lower level swimmers (the bottom 70% performances; L70) for minimum age (MA), progress (P) and best junior time (BJ). In order to identify the variables that contribute to reach the T30 group, a logistic regression (LR), stepwise LR and decision tree were applied. To analyze the effect of each variable separately, a simple LR (gross odds ratio) was performed. Ratio probabilities (OR) and 95% confidence intervals were calculated for each variable.

**Results:**

Swimmer’s BJ and P were higher in the T30 group (*p* < 0.000). The decision tree showed the greatest explanatory capacity for BJ, followed by P. The MA had a very low explanatory capacity and was not significant in the LR.

**Conclusion:**

Swimmers with exceptional junior performance times, or have a high rate of progress are more likely to be successful at the senior WCs.

## Introduction

Due to the increasing competition between nations for medals at major international events such as the World Championships (WCs) and Olympic Games (Bosscher et al., 2006), many national sporting organizations have invested their available resources more effectively by identifying talented athletes at younger ages (Vaeyens et al., 2009; Allen et al., 2015).

The primary aim of talent identification programs is to select athletes with potential who could later succeed in future (senior) international events (Allen et al., 2014). Those athletes then undergo targeted training through talent development programs (Boccia et al., 2017) based primarily on their age-related competition performance (Lloyd et al., 2015). In order to achieve success at senior levels, sporting organizations and coaches target talent development at youth levels.

Indeed, Svendsen et al. (2018) documented an increasing number of National governing bodies that have adopted long-term development models in an attempt to provide a structured approach to the training of their youth athletes.

Many studies have recently focused on junior performance levels and their impact on long-term potential (Sokolovas et al., 2006; Costa et al., 2010, 2011; Allen et al., 2015; Yustres et al., 2017; Kearney and Hayes, 2018). It has been suggested that a high level of junior race performance is a pre-requisite in becoming a successful senior athlete (Svendsen et al., 2018; Yustres et al., 2019).

If early performance achievement is a pre-requisite for senior elite success (Neeru et al., 2013; Green, 2015; Yustres et al., 2019), an important objective of long-term development models is to encourage swimmers to compete at an early stage in their career and sustain their involvement in the sport. If this is done correctly, the greater number of years competing in WC improves the subsequent positions achieved in the senior Championships (Yustres et al., 2019).

Athlete development has been described in many studies as an ascending scale and predicted improvements have been made using a linear model or pyramid (Barreiros et al., 2014; Green, 2015). Although reaching a final at the junior WC was not related to success in the senior WC (Yustres et al., 2017), participating (without reaching a final) in junior WC has a positive effect on superior performances in subsequent senior WC (Yustres et al., 2019). These results support the idea that swimming success is achieved through a gradual and progressive scale of talent development.

It would appear logical to track the progression of swimmers who have been successful at senior WC level in attempt to predict the pathway and requirements of swimmers for the future. Minimizing drop-out from junior to senior levels and accurate performance guidance models would be valuable in improving long term success (Costa et al., 2011; Allen et al., 2014). It has been suggested that the most significant reason for swimming drop-out in both genders and all age groups was “dissatisfaction/other priorities (e.g., education)” Monteiro et al. (2018). If a clearer predictive model was available, swimmers, coaches, and parents would have a better guide to develop their talent and improve satisfaction within the sport based on setting realistic expectations. It is likely that this planning would improve long-term performance and improve the retention of swimmers within the competitive system. One example which appears to be proving successful in nurturing junior and senior medal winning at International level is the sports system in the United Kingdom. It is now a requirement for national governing bodies of sport (including swimming) in the United Kingdom to have a sport-specific Long Term Athlete Development (LTAD) plan to receive state funding (Lang and Light, 2010).

It is suggested that the talent identification and development process in sport (including swimming) should hold three main components: Identification, Development and Follow-up. Respectively, these include athlete selection, optimal training content and a process of constant reflection and analysis to modify and improve the system based on current best practice (Morais et al., 2017). Indeed, the methods of assessment and the developmental stages are a critical in ensuring time efficiency and effectiveness of testing procedures for coaches, swimmers and sport scientists (Mitchell et al., 2018).

In order to determine the factors that could affect long-term progression, Larson et al. (2019) concluded that early specialization was not positively related to burnout and drop-out when analyzing the sporting background of 137 swimmers. Longitudinal studies such as this can indicate the likely speed of development and the methodical practice necessary to gain expertise. It also provides athletes, parents and coaches with better perspective regarding their likely future success and give more realistic career advice (Costa et al., 2011). Although designing an overarching model of swimming as a single sport is attractive, it would be more valuable if we were able identify the characteristics of the individual specialisms (such as stroke, distance, and gender) within this multi-event sport.

The development of a more detailed and accurate model would enhance our understanding of changes in performance across development stages and pathways to success. This would be beneficial for the wider sporting society, including governing bodies, administrators, parents and coaches, to enhance youth sport and setting realistic performance expectations (Shibli and Barrett, 2011; Tønnessen et al., 2015). In swimming, recent reviews of long-term development models and their content have been described within the literature (Allen et al., 2014; Dormehl et al., 2016). However, a retrospective examination of elite swimmers participating in the junior and senior WC and analysis of factors of predictive success have not been conducted.

Therefore, the main goal of this study was to create a performance progression model of elite swimmers racing in the WCs from 2006 to 2017 for all strokes and distances. Secondly, to identify the influence of annual ratios of progression, ages of peak performance and junior status on success at the senior WCs. We hypothesized that: (a) swimmers achieving better performances in the Junior WCs will be likely to reach a higher level in the senior WCs; (b) A higher annual progression rate improves the chance of success at the senior WCs.

## Materials and Methods

### Subjects and Design

This retrospective study was conducted in accordance with the declaration of Helsinki.

The authors have no conflicts of interest to disclose. The Castilla-La Mancha University Ethical Committee approved this research dated November 30th 2016 and since the data are based on publicly available resources, no informed consent was obtained. The International Swimming Federation (FINA) annually publishes WC results^1^. Thus, we retrieved all historical data from official results websites for the 2006, 2008, 2011, 2013, 2015, and 2017 Junior WCs and 2007, 2009, 2011, 2013, 2015, and 2016 senior WCs.

The database of 29031 entries contained records from 5878 swimmers after duplication and error removal. Senior World Championship swimmers totaled 3280 (55.8%), Category 1 (C1); 1893 (32.2%) only participated in the Junior WCs, Category 2 (C2); and 705 (12.0%) participated in both championships, Category 3 (C3).

The final, filtered database included only C1 and C3 swimmers and data recorded as mean (±standard deviation, SD) by distances, gender and swim strokes for a more appropriate standardization of the times. Each entry contains the following variables: race time, status [highest finishing position: final (3), semifinal (2), heats (1)], country, gender, age, swim stroke, distance, maintenance years (number of years that the swimmer has remained participating in WCs till the year of the WC in which they participate) and the year of competition. The distances analyzed were 200 and 400 m individual medleys; 50, 100, and 200 m backstroke/breaststroke/butterfly and 50, 100, 200, 400, 800, and 1500 m freestyle.

### Procedure

Times have been standardized by means of *Z*-time scores in order to compare swimmers’ times without influencing category, swim stroke, distance and gender. *Z*-time was then adjusted to include the annual best result of each swimmer categorized by gender, swim stroke, and distance.

(1) $$Z_{i\,j}=\frac{X_{i\,j}-{\bar{X}}_{i}}{\sigma_{i}}$$

where j = individual i = group by gender, swim stroke and distance.

(1) (dichotomous variable), those senior swimmers whose season’s best performance are within 30% of the competition best performance (T30) and the remaining 70% for those whose seasons’ best performance are in the lower 70% of the respective competition time (L70).

The following variables were analyzed for statistical significance: minimum age (MA): age in years in which they competed in their first competition; progress (P) in percent (%, an annual average of the interannual variations of standardized performances); A favorable progress is that where this average is negative and vice versa; best-time junior (BJ) in percent (%, the best-standardized performance at the junior competition).

Junior and Senior swimmers were divided separately by deciles (values of a variable that divides the distribution of the variable into ten groups with equal frequencies); the decile 1 (D1) is the top 10% best performances, decile 2 (D2) is the second 20% of performances etc.

### Statistical Analysis

Standard statistical analyses (mean, standard deviation, quartile, *T*-test, etc.) were applied in order to summarize quantitative characteristics in the study. A logistic regression (LR) was used to identify the most relevant aspects that discriminated between T30 and L70 and related these to their results in the junior WC and their progress to senior International swimmers. In order to analyze the effect of each variable separately, a simple LR (gross odds ratio) was performed. To identify the variables related to reach T30, a LR and a stepwise LR were calculated to reduce the Akaike coefficient (AIC).

(2) $$Y_{i}=\frac{e^{\alpha +\beta_{1}\,X_{1\,i}+\beta_{2}\,X_{2\,i}+\ldots +\beta_{k}\,X_{k\,i}}}{1+e^{\alpha +\beta_{1}\,X_{1\,i}+\beta_{2}\,X_{2\,i}+\ldots +\beta_{k}\,X_{k\,i}}}+\epsilon_{i}\mathrm{Logistic}\mathrm{reggression}\mathrm{model}$$

Where Y (T30) can take the values 0,1; representing the value 0 the absence of reaching T30, and the value 1 to reach T30. The X variables are explanatory variables. The relative variation of the advantage in reaching T30 with respect to L70 has changed proportionately when changing from any value xs to xs+1 of the variable Xs (Odd Ratio, OR).

Ratio probabilities (OR) and 95% confidence intervals were calculated for each case. In these multiple models, the fit to the model (McFadden test R2), the estimation of the coefficients associated with each explanatory variable and an estimation of the Nagelkerke determination coefficients were weighted. Non-parametric tests were also used to estimate the same model with a classification methodology based on decision trees. Both models were compared and additional conclusions were drawn from those already obtained with LR. A learning sample and a test sample were obtained after dividing the total sample. The learning sample was used to estimate both models and the test sample allowed the estimated models to be validated. All analyses were performed with the software R.

## Results

### Trajectories From Junior to Senior

**FIGURE 1:**
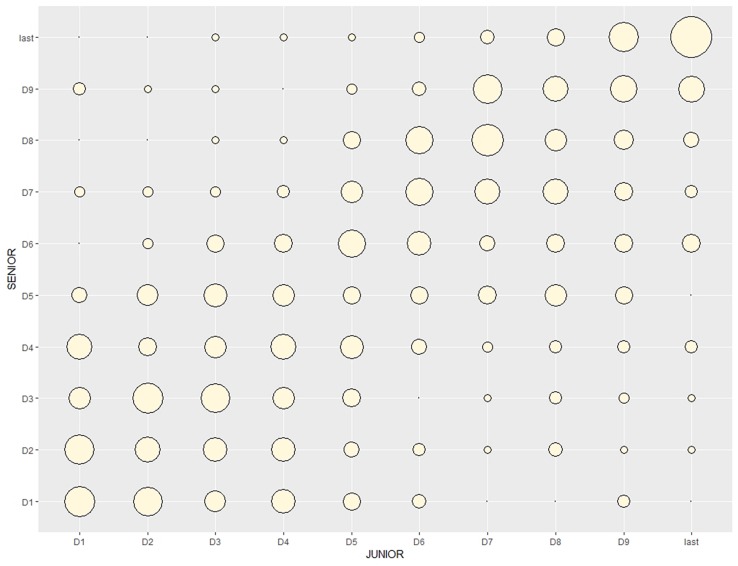
Improvement trajectories from junior to senior categories.

The effect of junior status on senior performances among swimmers from C3 found that 27.1% of swimmers who achieved D1 at the Junior WC, also did at Senior WC. In addition, 50.7% of D10 at the Junior WC were also classified in the D10 category at the Senior WC (Figure 1).

### Performance Progression Models

Mean ±SD age of the T30 swimmers was higher than L70 (20.4 ± 2.7 vs. 18.8 ± 2.4 years, *t* = 7.3660, *p* < 0.0001. The coefficients for the LR model derived from applying the stepwise method to reduce the Akaike AIC coefficient are reported in Table 1.

**TABLE 1:** Coefficients for the logistic regression (LR) model.

**Coefficients**	**Estimate**	**Std. Error**	***Z* value**	**Pr (>[z])**	**Or**	**Exp.loci.**	**Exp.upci.**
(Intercept)	−4.53	2.22	−2.04	0.04 ^∗^	0.01	0.00	0.83
MA	0.01	0.13	0.13	0.89	1.01	0.78	1.32
P	−0.06	0.00	−8.48	<2e-16 ^∗∗∗^	0.93	0.92	0.95
BJ	−0.07	0.00	−9.84	<2e-16 ^∗∗∗^	0.92	0.91	0.94

*MA, minimum age; P, progress; BJ, best-time junior. Significance codes: 0 “^∗∗∗^”, 0.001 “^∗∗^”, 0.01 “^∗^”, 0.05 “.”, 0.1 “ ” 1.*

Swimmers in the T30 and L70 groups first competed at similar ages (16.66 ± 1.12 vs. 16.39 ± 1.11). However, both P and BJ present significantly different mean values for both groups. Mean ±SD P was −38.7 ± 34.1 vs. −13.8 ± 35.9% for T30 and L70 groups, respectively (*p* < 0.01). There are also significant differences in the mean values of BJ. Mean ±SD BJ was −40.52 ± 36.16 vs. 6.14 ± 43.17 for T30 and L70 groups, respectively (*p* < 0.01).

The adjustment coefficient of McFadden (R2) was 0.51 and the Nagelkerke coefficient close to 0.7, suggesting an acceptable “degree of goodness” in the adjustment and therefore a model with good explanatory power. The confusion matrix showed an accuracy of approximately 84%, representing an acceptable degree of predictive capacity for the model.

### Explanatory Capacity of Variables

**FIGURE 2:**
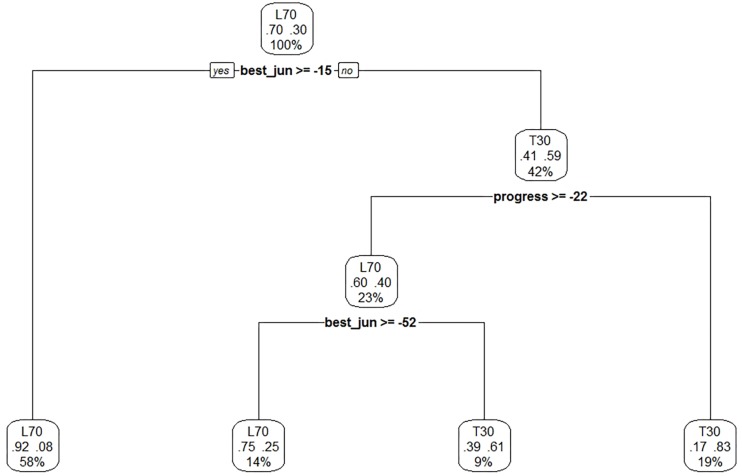
Decision Tree.

Deviation analysis (decision tree method) found the variable with the greatest explanatory capacity was BJ (59%). Secondly, P (31%) is of lower importance and MA (10%) barely has an explanatory capacity (Figure 2).

If the standardized BJ score is higher than −15%, there is a very high probability (92%) of not reaching the T30 status. In addition, if the BJ is better than −15% of the standardized score and the average annual progress is higher than −22%, there is a high probability (83%) of reaching T30 at the Senior WC. If the standardized score is higher than −52%, there is some chance of reaching T30 (61%), but if not, there is a 75% chance that swimmers will not reach T30 at the Senior WC (Table 2).

**TABLE 2:** Confusion matrix with the validation sample.

**Predicted**		
Actual	No	Yes
No	126	17
Yes	20	46
Accuracy	0.82	

## Discussion

The primary aim of the study was to create a performance progression model of elite competitors in the World Swimming Championships from 2006 to 2017 for all strokes and distances. The main findings of this study show that the T30 group are older than L70, despite a similar age at which they first compete at the senior WC. This suggests that a positive learning effect occurs and highlights the importance of competing in more than one WC in order to maximize success.

### Importance of Experience

The strong relationship between the number of years competing in the WC and the position achieved in senior WC races has been reported previously (Allen et al., 2014; Yustres et al., 2017, 2019). These studies showed that no significant differences were found between years of high-level competition YHLC for swimmers participating in CM Junior prior to their participation in CM Absolute and those directly participating in CM Absolute. Instead, they found a strong association between the variable position and YHLC, improving the positions as swimmers competed in a greater number of CM. Showing that a greater number of experiences at the international level will increase the chances of obtaining better returns in the Absolute CM.

Therefore, even greater importance should be given to the early specialization factor. It is not only a crucial factor as stated bellow to obtain the best positions in the absolute category, but also because it allows swimmers to participate in a greater number of CM, thus improving the positions received in CM Absolutes.

This suggests that governing bodies should establish a longer-term program of educational experiences based on selection for International competition and improvement. Future research should attempt to stablish the connection between the number of International competitions and performance level in order to identify an optimal number of experiences in advance of their target WC.

### Importance of Junior WC Performance

In this study, best junior time (decision tree 59%) showed the greatest explanatory capacity in predicting senior WC success. More than half (50.7%) of swimmers who achieved D10 in junior WC repeated this success at senior level. In addition, 27% of those who achieved the D1 level also did so at senior level. Prediction of senior success from junior level performance has also been found in other sports such as cycling (Svendsen et al., 2018).

In swimming, Yustres et al. (2017) found a strong association (*p* < 0.001) between senior WC position and the number of years spent participating at the WC. A higher number of WC representations was related to higher positions at the senior WC (odds = 1.85). Swimmers who participate in junior international events are more likely to achieve better positions at the senior WC (Yustres et al., 2019).

Conversely, Yustres et al. (2017) found that early specialization had no effect on the subsequent achievement of the senior WC finalists. This may be because early specialization is beneficial in some way but can be enhanced with a wider range of activities, educational experiences repeated exposure to International events. It is likely that a continual development based on addressing individual needs such as this will lead to a longer and more successful career than just focusing on junior performances in isolation.

The ratio in converting junior to senior success has been low in several studies (Barreiros et al., 2014; Durand-Bush and Salmela, 2002; Yustres et al., 2017, 2019). However, swimmers who have previously participated in Junior CM achieved higher positions than those participating only in the senior WC (Yustres et al., 2017, 2019). Therefore, despite a low conversion rate from junior to senior success, early specialization appears to be an important contributory factor to achieve better results at senior level.

### General Paths to Success

Development progression curves are typically non-linear Gulbin et al. (2013), with highly variable differences both within and between junior and senior competition standards (Yustres et al., 2017). In addition, Allen et al. (2014) found that most swimmers chosen to compete for the national junior squad did not progress continuously to senior representation. Performance prediction based on sub-elite male freestyle swimmers is not stable enough to ensure a degree of accuracy until 16 years of age (Costa et al., 2011).

Annual athlete performance improvement is an important factor in increasing the likelihood of reaching the highest international competition levels. Lower ranked junior athletes need a greater performance improvement rate than those athletes with higher rankings in order to catch up and increase the probability of winning a medal at senior levels (Pyne et al., 2001).

Hopkins et al. (1999) reported that an athlete in contention to win an international medal needs to improve their performance approximately one-half the typical race to-race fluctuation in performance (determined by standard deviation) to substantially increase their opportunity of medal success. Despite the studies that identify the variability in competitive performance (Hopkins et al., 1999), none have considered the annual performance progression in elite competitive swimmers. In this study, annual performance improvements were greater in T30 vs. L70 swimmers and provide a basis for swimmers and coaches to set plans with realistic goals for major International competitions.

### Study Limitations

Standardization of the swimming times provide some restrictions in order to illustrate more specific results by gender, swimming event or age. In addition, C2 swimmers (those who only participated in the junior WC) were not analyzed in our study because the main goal was to track the performance progression model of elite swimmers to the senior WC. Assessing the performance progress of C2 swimmers could provide coaches and organizations with valuable information about advice to give swimmer in their development that may help to minimize drop out and injures.

Future studies should consider a more performance-specific approach and show estimations of fluctuations and performance progressions of elite swimmers throughout their careers. Swimmers participating at the WCs are the ones used to establish the estimates with. Some other standards of athletes in other sports and swimmers may be analyzed in further studies.

### Summary

This is the first study to carry out a retrospective analysis of all swimmers participating in the WC since the first junior WC. It also reviews the most prominent models to provide useful information to swimming governing bodies and coaches to identify talent and develop optimal performance progressions for their swimmers.

Swimmers who have outstanding junior performance times or have higher rates of progress are more likely to achieve T30 positions at the senior WC. The ages at which swimmers compete in the WC for the first time does not influence T30 vs. L70 in the senior WC. Those swimmers who reach the T30 in junior competition are likely to reach T30 positions again in the senior championships. In addition, T30 athletes attain their personal best performances later than those swimmers from L70. Coaches, federations and High-Performance Centers could use this information in designing their talent identification and development programs.

## Conclusion

This study finds evidence to accept the hypothesis that: (a) swimmers achieving better performances at the junior WC will be more likely to reach a higher standard the senior WC; and (b) an optimal annual performance progression from junior WC positively affects the chances of success at the senior WC.

## Data Availability Statement

The data analyzed in this study are publicly available: http://www.fina.org/results?f[]=discipline_tid:45&f[]=year:2014&f[]=gms_event_name:12th%20FINA%20World%20Swimming%20Championships%20(25m)%202014.

## Author Contributions

IY, JG-R, and JC conceptualized, designed, and performed the experiments. IY, JG-R, JC, and FG-M analyzed and interpreted the data. IY, JG-R, MP, and FG-M edited and critically reviewed the manuscript. IY, JG-R, MP, and JC wrote the manuscript.

## Conflict of Interest

The authors declare that the research was conducted in the absence of any commercial or financial relationships that could be construed as a potential conflict of interest.

## Footnotes

1 http://www.fina.org/results
